# Role of Mucosal Inflammation in Eosinophilic Esophagitis: Review of the Literature

**DOI:** 10.5402/2011/468073

**Published:** 2010-11-25

**Authors:** Ghulamullah Shahzad, Paul Mustacchia, Marianne Frieri

**Affiliations:** Nassau University Medical Center, East Meadow, NY 11554, USA

## Abstract

Eosinophilic esophagitis (EE) is increasingly recognized in adults. It is an inflammatory disease of the esophageal mucosa, with variable presentation, unresponsive to acid suppression therapy. The diagnosis requires histological confirmation of intense eosinophilic infiltration on esophageal biopsy specimen, however exact criteria required to make a diagnosis of EE is still being debated and a clear differentiation from gastroesophageal reflux disease (GERD) is important. Allergen elimination or anti-inflammatory therapy may be effective in such patients. The imperfect diagnostic criteria for EE mandate an understanding of the immunology and the pathophysiology of the disease. It may facilitate the introduction of novel treatment modalities in an individual unresponsive to acid suppression therapy. This paper describes basic elements of the immune-mediated injury to the esophageal mucosa and management aspects to provide a better understanding of the condition.

## 1. Introduction

This paper will  summarize recent progress in the diagnosis and management of EE and discusses future research directions. Eosinophilic esophagitis was first reported in 1978 [1], but Attwood et al. [2] called attention to it as a distinct clinical condition in 1993.

Eosinophilic esophagitis (EE), a newly described clinicopathological condition, is being diagnosed with increasing frequency both in children and adults [3]. However, variability in the diagnostic criteria for EE still exist and requires further studies. 

Eosinophils with their reactive oxygen species, growth factors, and inflammatory mediators play an important role in the pathogenesis of EE. The release of these mediators in the esophagus can result in a cascade of events leading to inflammation of the esophageal mucosa. The expression of Th2 cytokine profile by these cells seems to be a key regulator of this disease process. 

There are demographic variables associated with EE. It is reported worldwide except in Africa. The condition is more common in males than females and more common in children than in other age groups. Caucasians are more commonly predisposed to this condition. The disease is equally prevalent in all socioeconomic strata and more common in families with a high IL-5 gene copy number and atopy [4]. It appears that the incidence of EE is on the rise in United States and it is being more commonly recognized [5]. Due to its association merely with benign complications, its prevalence is increasing worldwide [6, 7]. 

The complications that are recognized are strictures, motility disturbances, small caliber esophagus, and perforation. Perforation is most commonly associated with endoscopic procedures. The malignant potential of EE if any is unknown.

## 2. Pathophysiology

The resultant pathophysiological picture in EE primarily is due to an immune-mediated mechanism where food born and aeroallergens are proven to have a pivotal role [8]. Food allergies have been noted in 13%–25% with specific positive IgEs due to certain foods in 40% of adults [9, 10]. These allergies are thought to be mediated by activated CD4+T cells, found in the mesenteric lymph nodes [11]. T-cell transfer studies demonstrated that CCR6/CD196 was required to manifest allergic disease in the gastrointestinal tract [12]. The foods that are mainly implicated are high protein foods such as milk, eggs, soy beans, wheat, chicken, and nuts [13]. The swallowed aeroallergens after being transported from the lung to the esophagus via the mucociliary transport and inhaled allergens, can lead to mast cell activation in the esophagus and production of cytokines such as IL-4, IL-5, and IL-13. In addition, inhaled allergens such as dust mites also contribute to eczema which is associated with EE [14–16]. Additionally, in vivo experiments involving rodents have proven the role of IL-5 and eotaxin in airway sensitization and subsequent pathophysiology of EE [17]. 

Our division demonstrated the montelukast inhibited interleukin-5 mRNA expression and cysteinyl leukotriene production in ragweed and mite-stimulated peripheral blood mononuclear cells from patients with asthma [18]. Food and aeroallergens can be present in various forms such as intolerance, immediate or IgE-mediated and delayed type reactions [19]. Esophageal mucosa is initially exposed to allergen in a person with a history of atopy, and dendritic cells or langerhans cells will come in contact with the antigen engulfing it and the peptide fragments coated with MHC class II to make it foreign. These processed antigens will be recognized by the T cells resulting in a primary immune response and release of cytokines. IL-4 together with IL-2 stimulates B cells which results in production of IgE. Upon re-exposure to the allergen, the specific IgE bound mast cells degranulates its contents such as chemokines, histamine, and eosinophil chemotactic factors. Eosinophil migration and degranulation release eosinophil derived neurotoxin (EDN), IL-4, and IL-18 [20].

**Figure 1:**
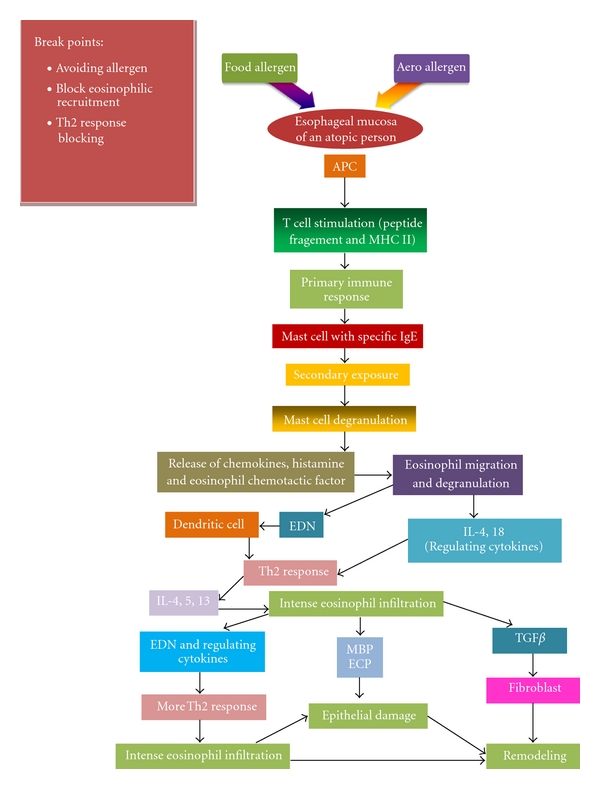
Schematic Diagram of pathophysiology of EE. APC: Antigen presenting cells, MBP: Major basic protein, EDN: Eosinophil-Derived Neurotoxin.

Upon further stimulation, EDN primes the dendritic cell to process the antigen and lymphocyte ensuing in a Th-2 type of cytokine response releasing IL-4, IL-5, and IL-13 [21]. These cytokines are the major products that stimulate mast cells and eosinophils resulting in eosinophil chemotaxis and inflammation [20]. Eosinophils then release their contents, including major basic protein, eosinophilic cationic protein, transforming growth factor-*β*, EDN, and regulating cytokines causing an intense inflammatory response that is followed by the healing process. This is a remodeling process which is similar to the lung in asthma [22, 23]. The continuous exposure to antigen and subsequent intense Th-2 response results in an inflamed esophagus that becomes infiltrated with large number of eosinophils and other inflammatory cells. As the inflammatory process continues fibroblasts will migrate and the reparatory process will have subsequent consequences such as small caliber esophagus, dysmotility, and stricture (Figure 1: Pathophysiology of EE).

## 3. Role of IL-5, IL-13, and Eotaxin

Chemokines play a central role in eosinophilic migration and inflammation in both tissues and blood. In knockout mice experiments, Hogan and his colleagues challenged allergen-sensitized mice with oral allergen, in the form of enteric-coated beads resulting in marked eosinophil accumulation in the blood and small intestine in the control mice. Eotaxin is a chemokine, constitutively expressed in the gastrointestinal tract. In eotaxin knockout (KO) mice, eosinophil recruitment into the mucosal lining was not seen with allergen stimulation and these mice developed enhanced eosinophil accumulation in the blood compared with control mice. Interestingly in IL-5 KO mice, allergen challenge promoted partial eosinophil accumulation into the small intestine with a decline in peripheral eosinophil levels. These results established an IL-5 independent and eotaxin-dependent mechanism of accumulation of gastrointestinal eosinophils provide a molecular base which explains the dichotomy between peripheral blood and tissue eosinophilia [24]. Eotaxin (specifically eotaxin-3) is found overexpressed in patients with EE [8]. The cytokine IL-13 has an established role in eosinophilic infiltration in diseases such as asthma and other allergies [25, 26].

 A variety of immune and nonimmune cells are known to produce IL-13 [27]. They have a prime role in eosinophil recruitment and eosinophil survival in EE, as it upregulates IgE and activates adhesion systems [28].

## 4. Clinical Features

The most common presenting symptoms in adults include dysphagia, food impaction, heartburn, and chest pain, while those in children include vomiting, regurgitation, and abdominal pain [29, 30].

## 5. Endoscopic Features

The feline, corrugated, or ringed esophagus is the classic endoscopic description of EE. Other endoscopic findings that are often described include linear furrows, white plagues, and a small lumen esophagus. Also, several reports have described a fragile, “crepe-paper”, esophageal mucosa [6, 7], which may also provide a clue for endoscopist. However, in many cases these endoscopic findings are absent, and a normal appearing esophagus is encountered. Therefore, in a patient with refractory symptoms, a high index of suspicion and multiple biopsies of the mucosa is essential in making the diagnosis.

## 6. Histopathology

The histopathologic features in EE are mainly due to the immunological insult caused by eosinophilic mediators. Normal mucosa of the esophageal lining is devoid of eosinophils. Under certain conditions such as infection and inflammation, inflammatory cell recruitment along with eosinophils occurs. However, certain stimuli such as food or aeroallergens will induce eosinophils via antigen presenting cells and mast cell-mediated pathways resulting in intramucosal and intramural infiltration. Due to the increasing number of eosinophilic load in the mucosa and deeper layers of the esophagus caused by immune-mediated mechanisms, it is suggested that the dysphagia in a person with normal mucosa must be carefully evaluated for EE. 

The classic feature of EE on endoscopic biopsy is superficial layering of eosinophils along with other inflammatory cells. It may also reveal eosinophilic microabscesses composed of more than three eosinophils, epithelial hyperplasia, edema of the mucosa, and inflammatory cells. In due course of time, a respiratory process with a fibroblasts remodeling of the epithelium takes place similar to remodeling in asthma [22, 31].

The definitive criteria to diagnose EE based on biopsy findings is not established due to the lack of availability of the results of randomized prospective studies. Lately, a case study of 41 patients suggested that eosinophil count greater than 15 cells/HPF is diagnostic [32]. As the distribution of eosinophils may not be uniform in the esophageal mucosa, it is recommended to take multiple biopsies from more than one place. There are no set criteria regarding the site of biopsy. Some studies suggest obtaining biopsy from proximal and distal portions of esophagus.

## 7. Allergic Association

Food allergy can be defined as an abnormal immunological response to food proteins that can cause an adverse clinical reaction [33]. The most common foods reported in eosinophilic esophagitis are eggs, milk, and fish, and there are many other food particles related in development of EE [30]. 

 Several other studies have reported a food association with EE, but these studies are lacking in adult population. In one pediatric study, children with EE were found to have more than 60% with food allergies [34]. In one large retrospective analysis, children with diagnosis of EE have more intense symptoms during summer or fall than in winter [35]. Environmental allergens and pollens are associated with EE. Fogg et al. reported a case of 21-year-old female presenting with diagnosis of EE and seasonal variation in biopsy proven eosinophils count [36].

Mishra et al. demonstrated that inhalation of Aspergillus causes eosinophilic esophagitis, and further described that mice showed eosinophils migration to esophagus after allergen was challenged [37]. In addition to sensitivity of food and seasonal allergies, a large series of patients were reviewed. In this study, out of 103 patients more than 73% with diagnosis of EE had positive family history of allergic diseases [38]. Previous pediatric as well as adult studies have revealed association of more than 70–80 percent patient population having concurrent diagnosis of different allergic disorder with EE [39, 40]. Children with food allergy to milk protein and eczema can have EE which can be missed in their diagnosis [41].

A prospective study of 31 patients by D. Simon and H. U. Simon in 19 patients had a history of allergic rhinitis or other allergic disease, which showed a strong coincidence of atopic disease and their role in pathogenesis of eosinophilic esophagitis [42]. The majority of patients with eosinophilic esophagitis have coexisting food and allergic diseases, and similar findings have been described in case reports, where two patients with food allergy, asthma and allergic rhinitis were associated with eosinophilic esophagitis [43].

## 8. Treatment Options

Dietary elimination, systemic and topical corticosteroids, leukotriene receptor antagonists and most recently, an anti-interleukin-5 monoclonal antibody have been used to treat EE. However, the best single possible therapy has not yet been defined. Available literature for possible therapy is either with case series or a very small clinical trial with poor clinical outcomes. 

### 8.1. Diet Therapy

Treatment approach in children and adults is somewhat different. Various dietary methods have been cited in the literature ranging from elemental diet to dietary elimination. Most of these studies suggest the positive role of diet therapy in providing symptomatic relief. A large randomized prospective study was conducted in patients with a diagnose of EE that were tested for food allergy with skin prick or atopy with a skin patch. In those subjects with a positive test result a restricted diet was applied for 4–8 weeks and a significant histological improvement of esophageal inflammation was documented in more than 70% of the study population [44].

Another approach was the introduction of an elemental diet in patients with a diagnosis of EE. In a small study by Kelly et al., twelve children diagnosed with EE were treated with a diet rich in amino acids which had a promising effect in the resolution of symptoms [45]. Yet, in another large series by Markowitz et al., there was complete histological and symptomatic resolution on elemental diet in EE subjects [46]. Many other clinical trials have been found with similar results, but long-term efficacy of treatment is yet to be described. The recurrence of symptoms with reintroduction of nonelemental foods is a major drawback in this study group. Moreover, these approaches have significant risk of nutritional deprivation and can lead to significant psychological burden on patients and their families [47]. Hence, a registered dietician may be helpful in the management of these patients [33].

### 8.2. Steroids

Use of steroid in inflammatory or allergic disease is not new. The role of steroids in EE patients is proven to be effective, however, its potential side effects limit its role in long term therapy. In a prospective cohort series, all patients were treated with oral steroids for 4 weeks and were followed with a clinical and histological evaluation before and after treatment. Patients in steroid group had a histological improvement by decrease in eosinophilic count on biopsy and 65% of 20 patients had complete resolution of symptoms. In spite of significant early improvement, majority of the patients relapsed after the withdrawal of steroid therapy and their role as a long-term management strategy is still unclear [48].

### 8.3. Montelukast

Eosinophilic stimulation generates a wide range of inflammatory cytokines including IL-1, IL-3, IL-4, IL-5, IL-13, and leukotrienes. Treatment success with montelukast is similar to steroids, with recurrence of disease upon withdrawal. In a study by Attwood et al., eight of their patients achieved complete remission of dysphagia after receiving montelukast. However, at the cost of side effects there was recurrence upon withdrawal in more than 70% of patients within 4 weeks of discontinuation of therapy [49]. In our study as referenced earlier montelukast inhibited interleukin-5 mRNA expression and cysteinyl leukotriene production in ragweed and mite-stimulated peripheral blood mononuclear cells from patients with asthma [18].

### 8.4. Anti-IL5 Therapy

This therapy has gained recent attention in scientific community as there is a potential role of IL-5 in pathogenesis of EE. In an open label randomized and placebo controlled phase II trial by Stein et al., humanized monoclonal antibody was tested in 4 adult subjects. The outcome was significant with a rapid clinical improvement and reduction of eosinophils in the esophagus and in blood [50]. 

In a pilot randomized placebo-controlled trial for safety and tolerability, 11 patients with severe EE who were unresponsive to steroids were evaluated. Histological analysis revealed a sound clinical improvement in the active group as compared to placebo group [51]. Mepolizumab, a well-tolerated novel monoclonal antibody seems to be clinically effective; however a large clinical trial is needed to produce robust result. Various isolated therapies with different agents such as purine analogue Anti-TNF therapy has been reported in the literature but these agents either have no effect or need large randomized clinical trials [52].

## 9. Discussion

EE is emerging as a global disease with complex pathogenesis. One of the biggest challenges in EE is to establish the cause of eosinophilic insult in organ where eosinophils are not naive. Since the awareness of this entity many studies tried to establish genetic and environmental factors which may be playing role in this complex entity. In one study patients with diagnosis of EE have elevated gene expression, particularly one gene called eotaxin-3, which plays significant role in the pathogenesis of EE. 

Although considerable advances are made in the understanding of human pathogenesis, the next challenge will be to understand the clear molecular mechanisms involved in EE disease expansion. Future studies may provide us new biomarker to differentiate EE from other esophageal disorders. The diagnostic criterion of EE based on endoscopic biopsy is still debatable and needs more refinement. The sites of the biopsy depending on the gross picture of the esophageal mucosa have not been established. Although some treatments are effective in EE, the molecular mechanisms involved in the remission have still not been established. The development of in vitro and in vivo models may help to dissect out the molecular mechanisms involved in remission or resistance to therapy. The overall goal is being able to molecularly classify patients as a function of their predicted response to treatment. Our current knowledge suggests that targeting the IL-13/eotaxin-3/CCR3 axis may be a promising therapy of EE.

**Figure 2:**
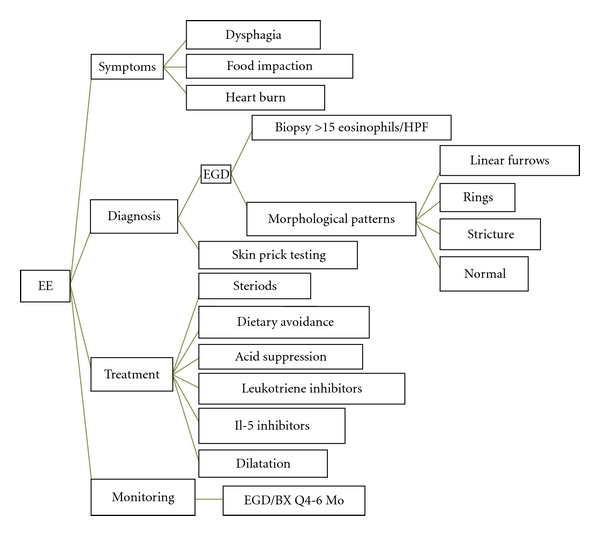
EE in a nut shell: Diagnosis and Treatment. EE: Eosinophilic esophagitis, BX: Biopsy, EGD: Esophagogastroduodenoscopy.

In summary, EE is the most common manifestation of the Eosinophilic Gastrointestinal Disease (EGID) spectrum, which appears to be increasing worldwide. The increase in prevalence suggests a need for more definitive diagnostic criteria and treatment (Figure 2). The pathophysiology of EE suggests the role of certain food or aeroallergens in a genetically susceptible individual. A multidisciplinary team involving a primary care physician, endoscopist, nutritionist, and allergist immunologist might be a superior approach to deal with this disease. More definitive research is indicated to further elucidate the role of eosinophilic mediators in order to plan treatment options such as diet and interleukin therapy. It is imperative that EE has to be considered in the differential diagnosis of treatment resistant GERD, as the dichotomy of the treatment modalities may result in early recovery of this condition and to avoid complications.
